# New Targets and New Technologies in the Treatment of Parkinson’s Disease: A Narrative Review

**DOI:** 10.3390/ijerph19148799

**Published:** 2022-07-20

**Authors:** Nicola Montemurro, Nelida Aliaga, Pablo Graff, Amanda Escribano, Jafeth Lizana

**Affiliations:** 1 Department of Neurosurgery, Azienda Ospedaliera Universitaria Pisana (AOUP), University of Pisa, 56100 Pisa, Italy; 2 Medicine Faculty, Austral University, Buenos Aires B1406, Argentina; nelida.aliaga.s@gmail.com (N.A.); amandaescriar@gmail.com (A.E.); 3 Functional Neurosurgery Program, Department of Neurosurgery, San Miguel Arcángel Hospital, Buenos Aires B1406, Argentina; pegraff@hotmail.com; 4 Department of Neurosurgery, Hospital Nacional Guillermo Almenara Irigoyen, Lima 07035, Peru; jafethlizana@gmail.com; 5 Medicine Faculty, Universidad Nacional Mayor de San Marcos, Lima 07035, Peru

**Keywords:** Parkinson’s disease, deep brain stimulation, new targets, subthalamic nucleus, functional neuroanatomy, new technologies, neurosurgery

## Abstract

Parkinson’s disease (PD) is a progressive neurodegenerative disease, whose main neuropathological finding is pars compacta degeneration due to the accumulation of Lewy bodies and Lewy neurites, and subsequent dopamine depletion. This leads to an increase in the activity of the subthalamic nucleus (STN) and the internal globus pallidus (GPi). Understanding functional anatomy is the key to understanding and developing new targets and new technologies that could potentially improve motor and non-motor symptoms in PD. Currently, the classical targets are insufficient to improve the entire wide spectrum of symptoms in PD (especially non-dopaminergic ones) and none are free of the side effects which are not only associated with the procedure, but with the targets themselves. The objective of this narrative review is to show new targets in DBS surgery as well as new technologies that are under study and have shown promising results to date. The aim is to give an overview of these new targets, as well as their limitations, and describe the current studies in this research field in order to review ongoing research that will probably become effective and routine treatments for PD in the near future.

## 1. Introduction

Parkinson’s disease (PD) is a chronic, progressive, and debilitating neurodegenerative disease that stands as the second most common neurodegenerative disease and affects about 1% of the population aged above 55 years old [1,2]. PD is the most common cause of “parkinsonism”, a syndrome manifested by rest tremor, rigidity, bradykinesia, and postural instability [3]. These four clinical presentations are commonly present; however, they do not occur long after the disease arises and therefore, they are not included in the Movement Disorder Society (MDS) diagnostic criteria for PD [4,5,6,7,8]. In PD the main neuropathological finding is substantia nigra pars compacta degeneration because of the accumulation of Lewy bodies and Lewy neurites [2,9] and subsequent dopamine depletion [10]. 

Deep brain stimulation (DBS) is a technique in which one or more multiple-contact electrodes are implanted in specific brain regions. These electrodes are connected to a subcutaneous implantable pulse generator from which the depolarization, re-polarization and conduction patterns of the neurons’ action potential are electrically modulated. Typically, high frequency stimulation (more than 100 Hz) mainly decreases the ipsilateral discharges from the subthalamic nucleus (STN), but there is also a decrease in contralateral STN nucleus firing [11,12,13,14]. Moreover, DBS is a three-dimensionally complex phenomenon, and consequently, a decrease in the action potentials in related nearby areas has been reported [15,16]. Even though there are paradoxical in vitro results published in the literature, it is probably because in vitro samples are disconnected from their afferents, so they do not have the chance for orthodromic stimulation [17,18]. However, aside from the DBS effects, it appears to be transient, with several articles suggesting that DBS as well as new technologies may induce long term changes in neuronal properties [19,20,21]. Neurochemical differences (GABA, glutamate, DA, cGMP, mGLUR, DR1/DR2) and induction of synaptic plasticity have also been observed in the target and in other related deep nuclei, structures, and the cortex itself [16,19,20,21,22,23].

Currently, the FDA (Food and Drug Administration) has approved modulation by DBS in the subthalamic nucleus (STN), the internal globus pallidus (GPi) and the ventralis intermedius nucleus of the thalamus (Vim). Targeting just these classical targets is insufficient for improving the entire wide spectrum of symptoms in PD (especially the non-dopaminergic ones), and none are free of the side effects which are associated not only with the procedure, but with the targets themselves. However, new targets and new technologies that could potentially improve the motor and non-motor symptoms of PD are currently proposed in the literature and are here discussed.

## 2. Overview of Basal Ganglia Functional Anatomy

**Figure 1:**
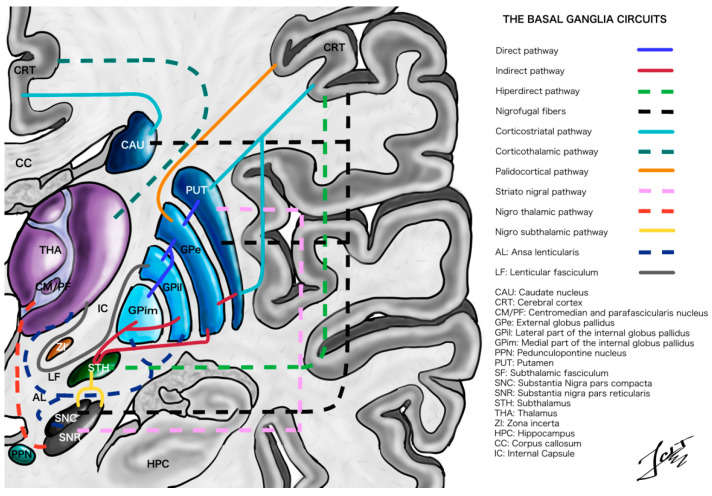
This figure shows a coronal section of the basal ganglia circuit with emphasis on its complex connections. Illustrated by J. Lizana.

The classical model of the basal ganglia includes both the direct and indirect pathways [24,25,26]. Both pathways are modulated by dopamine from the SNc (nigrofugal pathway) which sends projections to the posterior putamen, which in turn sends GABA projections back to the SNc. In addition, the motor cortex, the somatosensory cortex and the STN send glutamatergic fibers to the SNc [24,26,27]. However, there is evidence that the striatofugal pathways (direct and indirect) can no longer be considered dual, due to complex and extensive networks with advanced patterns of bridging collaterals (Figure 1) [28]. This provides straightforward evidence of the overlap and the delicate balance between both pathways [28,29]. Additionally, it is known that there are different functional regions in the striatum and the pallidum for the sensorimotor, associative, and limbic components [30]. For example, the head and the tail of the caudate have mainly associative fibers, while the putamen has mostly sensorimotor connections. Additionally, the ventral striatum has predominantly associative fibers and is also related to the limbic system [30,31]. However, in the end it is difficult to draw a boundary line between the dorsal and the ventral striatum, as well as between the functionality of each one [30].

**Figure 2:**
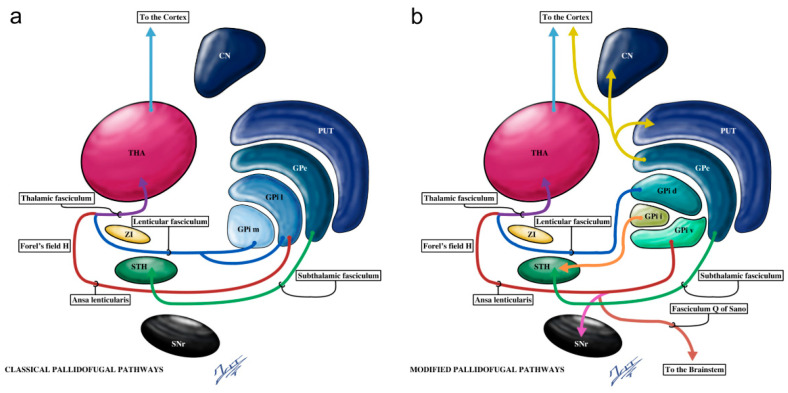
Figure (**a**) depicts the classical pallidofugal pathways, while Figure (**b**) illustrates some changes in the recent understanding of how the Globus pallidus communicates with other basal ganglia structures. Illustrated by J. Lizana.

The pallidofugal pathway is related to symptoms such as tremor and stiffness [32] and it is also known that 90% of cells that make up the GPi are motor neurons which connect to the PPN, ventral and CM/PF nuclei of the thalamus; however, there are 10% of limbic neurons that reach the lateral habenula [25]. Traditionally, it was believed that in the pallidofugal pathway the bipolar neurons at the lateral GPi would send their axons to the thalamus through the lenticular fasciculus and the ansa lenticularis, while neurons at the medial GPi would only send their axons through the lenticular fasciculus, and the GPe would send its efferents through the subthalamic fasciculus to reach the STN (Figure 2a) [25,33,34]. Nevertheless, there is evidence that the pallidofugal pathway fibers from the dorsal part of the GPi travel through the lenticular fasciculus, then pass between the STN and the ZI to finally reach the ventral part of the thalamus, while the fibers from the intermediate part of the GPi travel through the pallidum subthalamic tract and reach the lateral STN side [25,33,35]. Finally, the axons from the ventral part of the GPi travel through the ansa lenticularis then connect to the SN and continue to the brainstem through the fasciculus Q of Sano or the pallido tegmentalis, while other fibers of the ansa lenticularis turn dorsally to reach the thalamus through the thalamic fasciculus (Figure 2b) [35]. In the case of the GPe, there is a description of a direct projection to the frontal regions of the cerebral cortex, the caudate and the putamen [34,36].

**Figure 3:**
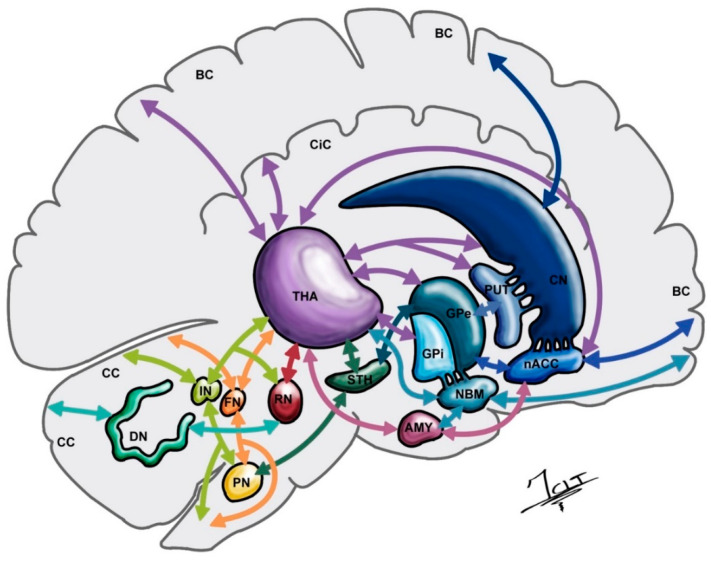
A sagittal section of the brain, trunk and cerebellum shows the complex connections between basal ganglia and cerebellum. AMY, Amygdala; BC, Brain cortex; CC, Cerebellar cortex; CiC, Cingulate cortex; CN, Caudate nucleus; DN, Dentate nucleus; FN, Fastigial nucleus; GPe, External globus pallidus; GPi, Internal globus pallidus; IN, Interpositus nucleus; NAcc, Nucleus accumbens; NBM, Nucleus basalis of Meynert; PN, Pontine nuclei; PUT, Putamen; RN, Red nucleus; STH, Subthalamus; THA, Thalamus. Illustrated by J. Lizana.

With regards to the cerebral cortex, its connections with the basal ganglia are also well known (Corticofugal pathway). For instance, the prefrontal cortex has connections with the caudate’s head; however, the dorsolateral prefrontal cortex and the pre-supplementary motor area are related to the anterior putamen [2,26]. Meanwhile, the limbic cortical areas that are connected to the ventral striatum and the motor cortex by the corticostriatal pathway, reach the posterior putamen [2,26,30]. The study of these pathways resulted in the identification of the glutamatergic hyperdirect cortico-subthalamo-pallidal circuit which plays a very important role in DBS procedures [24,37]. Furthermore, functional images have provided evidence of cerebellar activation in a wide variety of motor and non-motor processes [38,39,40,41]. It has been proved that a lot of cortical areas and basal ganglia nuclei, which are targets of cerebellar output, also project back onto the cerebellum (Figure 3) [40,42,43]. STN DBS may not only normalize the functional activity of the associative and limbic cerebellar cortices and decrease the activity of Purkinje cells with consequent disinhibition of the cerebellar nuclei, but could also improve some non-motor symptoms [40,44,45]. There is also evidence that STN DBS in dystonia, Tourette syndrome and PD results in decreased cerebellar hypermetabolism and symptom improvement [40]. Research in functional neuroanatomy has allowed critical advances in the development of new devices as well as the possibility of new targets.

## 3. New DBS Targets

### 3.1. Pedunculopontine Nucleus

The pontine peduncle nucleus (PPN) is a motor center of the trunk that is related to the initiation and modulation of the gait and receives afferents from the cortex, the central core, the trunk, and the medulla [46,47]. The PPN consists of the Cholinergic Pars Compacta (PPNc) and the Pars Dissipatus (glutamatergic and cholinergic) [47] and it is located medially to the middle lemniscus and laterally to the decussation of the superior cerebellar peduncle. Its rostral portion is posterior to the SN and the RRF, while its caudal pole meets the reticular formation, the cuneiform nucleus, and the locus coeruleus [48]. It has been observed that low-frequency stimulation of the PPN (bilateral or unilateral) inhibits the GABAergic neurons of this nucleus, which allows their activation and increases movement [47,49,50]. It is important to note that this nucleus may be involved in PD. However, the stimulation of this area may compromise adjacent structures such as the caudal portion of the mesencephalic reticular formation [47,48,49,51]. In many cases, DBS of the PPN has had good results on different aspects of the gait and appears to be a safe target [51]. Nevertheless, this effect is not uniform in all studies, nor in all patients [52,53,54].

### 3.2. Zona Incerta

The caudal zona incerta is a known target and is part of a region called the posterior subthalamic area, which is located posteromedial to the STN, inferior to the thalamus and lateral to the red nucleus [9]. It has been observed in studies carried out in patients with STN-DBS, that stimulation of this area and the adjacent ones (pallidothalamic fibers) improves the results, which contrasts with patients where only the STN is stimulated, thus developing interest in the surrounding structures (the zona incerta) as a possible therapeutic target since it is a unique GABAergic link between the basal ganglia output nuclei, the cerebello-thalamo-cortical loop and the brainstem nuclei [55,56,57,58].

Due to their interconnections with the basal ganglia and the cerebellum (Figure 1), stimulation of the zona incerta (incerto-thalamic fibers) and the bundles that cross this area (the nigrothalamic fibers, the incerto-pallidal bundle, the pallidal-thalamic pathway and the cerebellar-thalamic tract) improves, importantly, symptoms such as essential voice tremor compared to STN stimulation [46,59], and also results in decreases in bradykinesia, rigidity, ataxia and abnormal muscle activities [60,61]. Moreover, stimulating the ZI and adjacent areas decreases the required dose, and reduces the adverse effects, of dopaminergic medication [62]. Other points in favor of this target are that it is relatively easy for neurosurgeons to leave the deep ventral DBS contact in the caudal ZI, and that long term studies have shown that the ZI does not develop tolerance; however, Vim DBS did provide better outcomes [61,63]. However, it is necessary to highlight that it can cause or exacerbate dyskinesia, dysarthria, and can also alter pleasure-seeking behavior [60,64]. There are limitations to these findings with regards to it being a new target so it is a procedure rarely performed and with few studies on the subject, as such, we cannot define conclusions [61,63].

### 3.3. Thalamic CM/Pf Complex

The thalamus as a target (Vim) for DBS was the first to be studied and applied in clinical practice and demonstrated adequate control of the tremor at rest [65]. The centromedian/parafascicular complex (CM/Pf) is located in the posterior intralaminar thalamus [66,67]. It is an important center for sensory, limbic and motor processing association due to its extensive connections with the cortex, the basal ganglia and the brainstem [66]. It has been observed in patients whose dyskinesia improves that the thalamic electrode is closer to the CM/Pf than to the Vim [67,68]. Similarly, clinical improvement has been highlighted in tremor and freezing of gait, motivating interest in its role in DBS surgery [69,70]. Although it does not affect extrapyramidal symptoms with the same intensity as DBS of the STN, it seems to be a better target than the Vim and it opens up the possibility of a synergistic and complementary effect with other targets for patients with difficult-to-control symptoms [67,69,70,71].

### 3.4. Substantia Nigra Pars Reticulata (SNr)

The midbrain locomotor region is a functional territory that appears to be comprised of both the cuneiform nucleus and part of the PPN and is directly influenced by GABAergic nigro-tegmental projections from the SNr in addition to the supplemental motor cortex [72,73]. This relationship of the SNr with the ponto-mesencephalic and spinal structures motivated experimental studies where it was observed that both its unilateral and bilateral stimulation (and in many of these cases combining its effect with the stimulation of the STN), could favor the improvement of axial symptoms related to the onset of gait in patients with PD [72,74]. Furthermore, an improvement has also been noted in the axial symptom subsection of the UPDRS scale, thus constituting a potential tool for the treatment of postural instability and freezing of the gait [73,75,76]. However, psychiatric side effects (acute depression, hypomania and mania) have been reported in a variable way and are possibly related to its limbic connections [73].

### 3.5. Multitarget

Multitarget therapy is a novel and interesting option that involves more than one anatomical objective for patients in whom the most common targets such as STN or GPi, by themselves, are insufficient for controlling the symptoms of the disease, mostly, non-dopaminergic symptoms such as gait, balance impairment and cognitive decline [46,77,78]. As detailed in the previous sections, the use of new targets such as the PPN, the ZI, the SNr and the thalamus for DBS is under investigation [54]. These options are open to different therapeutic scenarios for the management of complex cases. If their effectiveness is demonstrated, it is most likely that they will not replace the classical targets but will be used as adjuvants in selected cases [79]. Similarly, it is possible to combine the stimulation of two classical targets such as the STN and the GPi to obtain better results [80]. The development of multitarget therapy is based on the improvement of surgical times, technological progress and the clinical benefits associated with biochemical changes in areas other than the STN for the legitimate control of clinical signs that cannot be controlled with classical DBS targets [70,80]. Candidates for multitarget therapy are patients with involuntary movements who are reluctant to decrease their dose of drugs; patients with severe tremor whose response to STN-DBS progressively decreases; patients freezing in ON periods; and even patients with mild cognitive impairment whose eligibility for STN-DBS may be in doubt [80].

One of the advantages of GPi/STN-DBS in PD is that it offers greater control of neuropsychiatric symptoms. [46,70] Currently, a GPi/STN-DBS strategy is more than an eligible approach for those cases where there is profound and medically refractory functional impairment [78,81]. GPi/STN-DBS has shown favorable outcomes; however, its effect has been seen to decrease over time. For this reason, more evidence is needed to fully understand the advantages and limitations of this dual stimulation [78]. Treating multiple targets with DBS is limited by its higher costs, the prolonged inconvenience of surgery due to its slower result, greater probability of complications, and the requirement of multidisciplinary and highly specialized healthcare staff for peri- and post-operative management [70,77]. This review is susceptible to changes through time, as technology and science become more advanced and new research will be reported in the near future.

## 4. Non-Invasive and Minimally Invasive Treatment

### 4.1. Endovascular Approach

**Figure 4:**
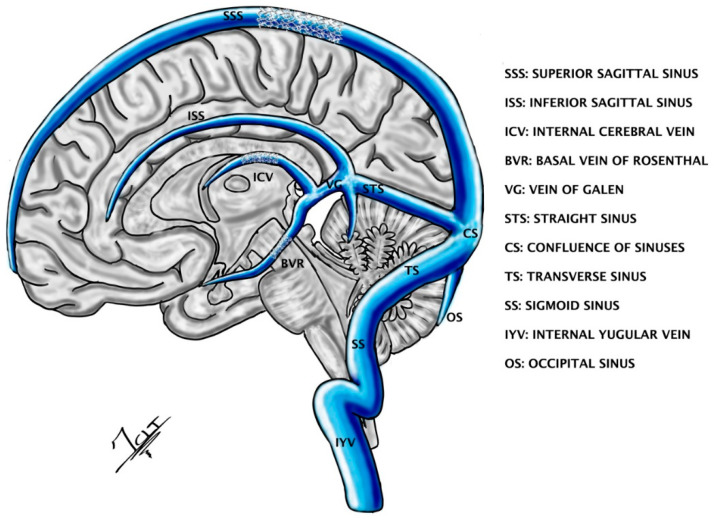
This figure shows some theoretical locations (superior sagittal sinus, internal cerebral vein and basal vein of Rosenthal) of STENTrodes which can be placed by the endovascular transvenous approach to stimulate the cortex, thalamus or brainstem nuclei. Illustrated by J. Lizana.

The use of endovascular devices (with cable or without cable) with the ability to record brain activity and stimulate adjacent tissue (STENTrodes) has emerged as an alternative among mechanisms of deep stimulation with minimal invasion for the treatment of PD, essential tremor, epilepsy, severe paralysis and many other diseases [82,83]. There is a wide variety of catheters, guide catheters, microcatheters and micro guides, among others, which facilitate the release of the devices in the desired location [82,84]. The endovascular routes for the release of the device can be by a transarterial or transvenous route [83]. However, the anatomical variations of the vascular structures are a limitation for the adequate positioning of the device, since precise planning for the identification of an accessible vascular structure (vessels with a diameter greater than 1 mm are more accessible) closest to the target is essential. Angiography and MRI protocols have taken on a greater relevance for the elaboration of probabilistic maps in order to improve the accuracy of the device placement [83,85,86]. Only some structures such as the nucleus accumbens, fornix, subcallosal cortex of the cingulum, dentato-rubro-thalamic tract, subthalamus and pontine peduncle nucleus, among others, are susceptible to stimulation by this route due to their vascular proximity [83,87] (Figure 4). It should be noted that the possibility of performing the procedure with the patient awake allows for evaluation of the procedure and observing whether the result is the desired one [88]. Although endovascular therapy has an encouraging future due to new and continuous advances, it must be considered that there are potential side effects depending on the location of the device, ranging from psychiatric disorders (due to proximity to the limbic system) to autonomic problems (due to proximity to the hypothalamus), and also those of the procedure such as intracranial hemorrhage and arterial or venous thrombosis by the intraluminal device (with SHIELD technology the thrombogenicity of the devices is reduced) [82,83,87,89].

### 4.2. Non-Invasive Transcranial Stimulation

Non-invasive transcranial stimulation is based on beta oscillatory activity (14–35 Hz anti-kinetic activity) that occurs at the level of the motor cortex and its connections with the basal ganglia (predominantly with the subthalamus and the GPe) [90,91,92]. These connections are exacerbated (excessive beta synchronization) in patients with PD [91,92,93,94]. This abnormal activity occurs mainly during involuntary tonic contractions and decreases with voluntary motor activity (beta desynchronization), as well as with pharmacological and surgical treatment [91,92,93,94].

Currently, there are two techniques known as “Non-invasive transcranial stimulation”: The first one, Transcranial Current Magnetic Stimulation (TMS) and the second one, Transcranial Current Stimulation (tCS) [95,96]. Both have proved to have a good efficacy rate in the modulation of cerebral activity [95,96,97]. One of the advantages of this non-invasive technology over DBS is that it can target cortical and subcortical structures; therefore, it can reach remote locations from the stimulation site [96,98]. It should also be mentioned that DBS is an invasive procedure, consequently, it goes hand in hand with some complications, such as infection, limited duration of electrical components, neural immune system reactions, and the need for periodic battery replacement [99,100,101,102]. One of the limitations of TMS is that this device is large and heavy, whereas tDCS is light and small, which makes it more feasible since it is a treatment that does not require hospitalization and the sessions can be performed at the patient’s home [97,103]. Cost is another big difference, with TMS systems costing between $20,000 and $100,000, while tDCS devices have prices ranging from $400 to $10,000 [95,97,104].

#### 4.2.1. Transcranial Magnetic Stimulation (TMS)

TMS is a well-tolerated and painless brain stimulation technique in which a strong and rapidly changing electromagnetic field is generated [95,97,103,105]. This electromagnetic field induces strong and short-lived electrical currents, which initiate action potentials in both the cortex and subcortical white matter [96,97,105]. The application of repetitive transcranial magnetic stimulation (rTMS) is able to induce lasting changes in cortical excitability, which represents a promising tool against neuropsychiatric alterations and motor symptoms in PD [98,104,106]. Unlike TMS, rTMS appears to be more effective in stimulating the association cortex [107]. Presently, TMS has a larger number of published studies demonstrating its safety in terms of effects on brain anatomy and biochemistry, than does tDCS [97,105]. Other studies being conducted, which link rTMS with an improvement in non-motor symptoms in PD such as cognitive dysfunction, speech difficulty and depression, show mixed results [105,107]. However, its greatest risk is the induction of seizures [108]. Preclinical data on the safety of TMS is still very limited, and rTMS protocols are needed to use this technology as a routine treatment for PD [95,98].

#### 4.2.2. Transcranial Current Stimulation—Transcranial Direct Current Stimulation (tDCS)

TCS has re-emerged as a form of non-invasive modulation of spontaneous neuronal activity by applying a weak electrical current (1–2 mA) through the placement of two or more electrodes on the scalp, allowing regulation of neuronal membrane potentials (changes in discharge probability), changes in neurotransmitters, glia, and micro vessels [109]. In other words, it provides neuronal plasticity, shown by the generation of long-term synaptic potentiation (objectified by the increase in brain derived neurotrophic factor (BDNF) secretion and TrkB activation) when combined with repetitive low-frequency synaptic activation [110]. Transcranial direct current stimulation (tDCS) affects the motor symptoms of the disease, with the most prominent results related to rehabilitation. However, its usefulness is limited due to its weak effects, high variability, and lack of consensus in protocols, with medication status being a key cofounder in determining the level of efficacy [111].

#### 4.2.3. Transcranial Current Stimulation—Transcranial Stimulation with Alternating Current (tACS)

Recent innovations in transcranial alternating current stimulation (tACS) offer new areas of research [111]. This is a variant of direct cranial stimulation and consists of the interference of sinus waves for the modulation of brain oscillations (physiological brain activity in an area) [109,111]. It has been observed that tACS has a different effect on healthy people compared to those with PD due to its effect on beta synchronization [92,112]. tACS seems to produce an improvement in motor symptoms in people with PD, which is further enhanced when associated with a closed loop system [92]. Furthermore, when this device stimulates the cerebellum, it also results in the modulation of parkinsonian tremor [113]. In addition to this, there is evidence that, like tDCS, it not only modulates brain activity, but also seems to generate cortical neuronal plasticity [114,115,116].

### 4.3. Non-Invasive Focused Ultrasound

Magnetic resonance-guided focused ultrasound (MRgFUS) is a novel, non-invasive procedure for symptomatic treatment of PD [117,118]. Two types of focused ultrasound therapy are recognized. The first one is called non-ablative: this technology has potential uses in pain neuromodulation, epilepsy, and is also seen as a new strategy to allow the passage of medication through the BBB, for instance, biological-genetic treatment in PD [117,119]. The second one is ablative ultrasound, in which two varieties are differentiated according to the frequency used. High intensity focused ultrasound (HIFU) produces thermal ablation in small brain targeted areas (therapeutic sonication) [118,120]. This technology enables accurate targeting by using a real-time MRI for morphological and thermal monitoring [120,121]. On the other hand, ablation at low frequencies is due to a fast change in the targeted tissue pressure, forming gas–vapor filled cavities. These cavitation bubbles oscillate at large amplitudes and exert shear stresses on the surrounding tissue, causing the mechanical cell membranes to tear (histotripsy) [119,122,123].

The use of HIFU in the Vim nucleus has been approved by the FDA for the treatment of tremor in patients with PD [117,120,124]. Candidate patients for Vim ablation are those with very asymmetric symptoms, who are not able to have or do not want surgery or implants, but does not including patients with refractory tremor. Exclusion criteria are severe bilateral axial symptoms, severe dyskinesia, cognitive impairment, or psychiatric illness [117]. Although unilateral ablation of the Vim seems to be effective, it has transitory side effects such as weakness, gait, and speech disturbances [120]. The STN is a potential target for MRgFUS; however, due to few studies being available, it cannot be considered as strong evidence. This procedure has a high rate of adverse effects, with gait instability being the most frequent; however, all are mostly transient [117,120,125]. Similarly, the results of GPi ablation in patients with PD is little but encouraging due to the effects its ablation has on dyskinesias, as well as the improvement of PD symptoms in off-L-dopa state patients [117,126]. Furthermore, challenges lie ahead in treating this target due to its proximity to the optic nerve and being able to correctly target the ultrasound rays on the treatment area [118,127].

The advantages of MRgFUS over DBS are that this non-invasive technology does not carry any risk of infection or hardware failure, and post-operative programming is not required as the effect is achieved immediately at the end of the procedure [120,128]. What is more, this procedure does not include any kind of brain penetration, therefore, it does not have the complications associated with foreign object implantation [120,129,130]. Gamma knife use also effectively suppresses tremor but is limited by the latent effects of radiation and the inability to target intraoperatively [130]. Nevertheless, some disadvantages of MRgFUS lie in its local effects, such as headache, scalp burns, and bone necrosis [123].

## 5. Regenerative Medicine in Parkinson Disease

Currently, none of the pharmacological or surgical treatments can cure or modify the neurodegeneration process that occurs in PD [131,132]. Furthermore, it is known that the current management of PD is insufficient and costs billions to the world economy. In the USA alone, the cost of PD is 35 billion annually and it is estimated that by stopping its progression, the economic benefit would be almost 450,000 dollars per patient [133]. Regenerative medicine has been progressively developed in recent decades with the aim of reconstructing and restoring the functionality of highly sophisticated neural circuits (connectome) that are lost in PD [131,132,134,135]. Technological progress in cell engineering, cell programming, immunomodulation, tissue engineering, biomaterials, gene therapy, and stem cells has allowed the development of several studies that project a promising future in this type of biological therapy [136,137,138,139,140].

### 5.1. Gene, Cell, and Tissue Regenerative Therapies

Autologous or heterologous cell transplantation with neural differentiation potential has shown satisfactory results in animals and humans [136,141,142]. Different cell types can be divided into these groups: fetal mesencephalic cells, adrenal medullary cells, carotid body cells, sympathetic ganglia cells, and retinal pigmentary epithelial cells [142,143]. Human fetal mesencephalic tissue grafts are the ones with the most evidence and the greatest success (documented improvement of motor symptoms) to date [144,145,146]. The objectives of this type of therapy are the survival of dopaminergic cells, afferent and efferent synaptic integration, and adequate and controlled dopamine release, in addition to the clinical improvement of patients [136,141,147]. There are reports of patients in whom the presence of transplanted cells has been demonstrated up to 24 years later; however, their potential susceptibility to degeneration is still not clear [145,148]. The problems with the clinical studies involving fetal mesencephalic cells include: the heterogeneity of the cells, in most cases the transplant has not been performed in the SNc region but in the striatum, there is a well-known limitation in the availability of fetal tissue, cells do not always differentiate as expected, it has been observed that the grafts are not made up purely of dopaminergic cells, this kind of procedure often requires immunosuppression, and there are underlying ethical considerations [142,149,150,151]. Another risk in the use of stem cells is the neoformation of tumors and the development of graft-induced dyskinesia (GID) [152,153,154].

There are other sources of cells with potential dopaminergic differentiation such as embryonic stem cells (ESC), induced pluripotential stem cells (iPSC), neural precursor cells (NPC), mesenchymal stem cells (MSC), and direct neuronal reprogramming [152,155,156]. ESCs come from early-stage embryos, which implies ethical problems, limited availability, and they have a significant risk of tumor development [152]. Knowledge of cell de-differentiation techniques as well as advances in neurodevelopment have allowed the generation of iPSCs with greater similarity to dopaminergic neurons and with a lower oncological risk [156,157]. NSC can be induced from iPSC and because of their limited differentiation capability they have less oncogenic likelihood [158]. On the other hand, MSCs have a more limiting potential for neural differentiation but have an immunomodulatory effect that counteracts neurodegeneration [159]. Problems with earlier grafts have generated an increased interest in neuronal reprogramming, in which virus vectors, microRNAs, or transcription factors can be used to alter the gene expression in astrocytes in order to generate dopaminergic neurons [138,140].

Despite advances in cell restoration, there is a gap to recover the lost functionality, that is, the development of new and appropriate neuronal circuits which maintain the dopaminergic function [139,160,161,162,163]. Axonal growth over long distances, its directionality, and the reinnervation of the correct target continue to be a matter of investigation [160,164,165,166,167]. Nowadays, chemoattractant factors are being used in the striatum to promote the restructuring of the nigrostriatal pathway [165]. Other strategies include the use of embryonic striatal tissue, renal tissue, kainate injections, and overexpression of GDNF (glial cell line-derived neurotrophic factor) in the striatum by virus vectors [168,169]. The development of new micro biomaterials has allowed the creation of scaffolds for grafts, that not only protect and promote the graft growth but can also be implanted in the form of neural networks that stimulate the nigrostriatal pathway and favor its restoration [139,160,170]. Finally, there is a potential risk that any cell or tissue engineering strategy may succumb to the underlying pathology and degenerate in a similar way to the original tissue, so developing new grafts resistant to different stress factors is a new objective [151,171,172].

### 5.2. Optogenetic Therapy

The lack of control over the delimitation of the stimulated area can lead to undesirable results [173,174]. Due to this problem, optogenetic therapy has emerged as an interesting option to avoid side effects due to the stimulation of structures adjacent to the target [175]. This therapy consists of the use of genetically altered viruses with the aim of modifying neuronal genes (the gene for an ultra-fast opsin called Chronos is packaged in the adeno-associated virus type V with a CaMKII promoter for a local effect on the subthalamic nucleus) and making them susceptible to excitation or inhibition by light, so that the desired effect is generated in a more controlled way in each area [171,172,173,174,175]. Optogenetic therapy opens a way for us to understand synaptic and circuit properties on a mechanistic level [176,177]. Favorable results in several animal studies lead us to believe that this therapy could be an important research tool for future DBS-based therapies, leading to symptoms being resolved, and not masking them [174,178]. However, to date this technique is not yet feasible in humans and suffers from other limitations such as light stimulation parameters (duration, frequency, and intensity of stimulation) which must be managed carefully to avoid problems such as depolarization blocks or rebound excitation [177,179,180,181]. We hope that one day this could be a viable treatment for movement disorders.

## 6. Discussion and Conclusions

Research into functional anatomy as well as clinical trials have allowed a better understanding of the basis, benefits, and limitations of DBS procedures [24,26,38]. The most common scope of studies in PD is directed towards motor symptoms [132]. However, non-motor symptoms are as relevant as these but less studied and, due to their great impact on quality of life, they require more intensive research [73,132]. Currently, progress in the development of devices is reflected in increasingly personalized treatments and the approach of new targets [46,77]. Although most of these targets require more evidence for their widespread use, they open the possibility of being associated with classical targets to achieve better symptom control [79,80]. One of the main disadvantages of DBS is the high cost involved while newer devices appear to be cheaper [92,104,133]. In addition, several of these new devices offer the advantage of being minimally invasive or non-invasive at all [85,87,105].

Accelerated advances in biological therapy, gene therapy, cell engineering, tissue engineering, and biomaterials have made it possible to propose new therapeutic options with the potential to modify the course of the disease [134,140,160]. Even though several studies have shown encouraging long-term results with the use of embryonic or fetal human cells, the limitations and risks of this type of graft have motivated the development of techniques for the induction of new cells with dopaminergic potential [151,156,157]. Nevertheless, we must consider that both the management of fetal or embryonic tissue, as well as genetic manipulation at the cellular or viral level, entail ethical considerations and risks [152,155,169]. The development and use of biomaterials in the peripheral nervous system is currently quite widespread; however, the central nervous system constitutes a more complex area for their use. The accuracy in the design, its microstructure that tries to reproduce the neural circuits, and the advantages shown in the studies are encouraging [164,169]. Most current studies are focused on technological advances, but this should not be separated from the study of functional micro neuroanatomy, since the integration and deepening of both will allow for better results.

There is parallel improvement in regenerative and non-regenerative treatment. Non-regenerative treatment seeks to improve the performance, invasiveness issues, and duration of its devices [46,77,96]. However, it has not been shown to affect the disease progression [118]. Merely stopping its progression would generate a major impact, but current initiatives go beyond that and seek to regenerate the tissue and lost connections, that is, to cure it [133,160]. As described, the new therapeutic options also have limitations and problems, but under the same logic of multitarget treatment, with the wide range of current options, studies of multimodal or hybrid management in PD should be considered. We believe that the treatment of PD will no longer be symptomatic related in due time and that the future directions of surgical management of PD are towards regenerative neurosurgery. We have described the current studies and ongoing research in PD in order to review the treatments that may become effective and safe in the near future for the treatment of PD.
